# Cdc25 and Wee1: analogous opposites?

**DOI:** 10.1186/1747-1028-2-12

**Published:** 2007-05-04

**Authors:** Jennifer A Perry, Sally Kornbluth

**Affiliations:** 1 Department of Pharmacology and Cancer Biology, Duke University Medical Center, Durham, North Carolina, 27710, USA

## Abstract

Movement through the cell cycle is controlled by the temporally and spatially ordered activation of cyclin-dependent kinases paired with their respective cyclin binding partners. Cell cycle events occur in a stepwise fashion and are monitored by molecular surveillance systems to ensure that each cell cycle process is appropriately completed before subsequent events are initiated. Cells prevent entry into mitosis while DNA replication is ongoing, or if DNA is damaged, via checkpoint mechanisms that inhibit the activators and activate the inhibitors of mitosis, Cdc25 and Wee1, respectively. Once DNA replication has been faithfully completed, Cdc2/Cyclin B is swiftly activated for a timely transition from interphase into mitosis. This sharp transition is propagated through both positive and negative feedback loops that impinge upon Cdc25 and Wee1 to ensure that Cdc2/Cyclin B is fully activated. Recent reports from a number of laboratories have revealed a remarkably complex network of kinases and phosphatases that coordinately control Cdc25 and Wee1, thereby precisely regulating the transition into mitosis. Although not all factors that inhibit Cdc25 have been shown to activate Wee1 and vice versa, a number of regulatory modules are clearly shared in common. Thus, studies on either the Cdc25 or Wee1-regulatory arm of the mitotic control pathway should continue to shed light on how both arms are coordinated to smoothly regulate mitotic entry.

## Background

Entry into mitosis is driven by the activity of the cell cycle regulatory kinase, Cdc2/Cyclin B, which oscillates throughout the cell cycle, peaking in mitosis and dropping during interphase. This precise temporal regulation is ensured by the coordinate action of positive and negative regulators of Cdc2 catalytic activity and localization. During interphase, once Cyclin B synthesis has begun, the newly formed Cdc2/Cyclin B complexes are kept inactive by phosphorylation on Cdc2 at two residues, Thr14 and Tyr15. These phosphorylations are catalyzed by the Myt1 and Wee1 kinases, which are located at cytoplasmic membranes and within the nucleus, respectively. At the G2/M transition, Myt1 and Wee1 are inactivated while the dual specificity phosphatase, Cdc25, is activated. Cdc25 dephosphorylates Thr14 and Tyr15, allowing for activation of the Cdc2/Cyclin B complex and entry into mitosis [1,2].

Just as Cdc2/Cyclin B activation is highly regulated, both Wee1 and Cdc25 activity are tightly controlled through the cell cycle. Interestingly, in considering what we know currently about the regulation of Wee1 and Cdc25, it appears that these two molecules are similarly regulated, though their activities oscillate in opposition to one another, consistent with their respective roles in inhibiting or activating mitotic entry. Both are phosphorylated and bind to 14-3-3 during interphase; both are controlled directly by other mitotic kinases, including Polo-like kinases and Cdc2/Cyclin B itself. Integrating data from yeast, Xenopus, and mammalian cells over the last decade, we can now take a comprehensive look at how cells coordinate the opposing actions of Wee1 and Cdc25 via post-translational modifications in order to regulate Cdc2/Cyclin B and entry into mitosis.

### Cdc25: background

The Cdc25 family of phosphatases is responsible for dephosphorylating inhibitory Tyr and Thr residues on cyclin-dependent kinases in order to promote their activation. Three isoforms (A, B and C) have been identified in mammals while only two isoforms (A and C) have been characterized in Xenopus. Traditionally, mammalian Cdc25A was classified as the G1/S-promoting phosphatase, while Cdc25B and Cdc25C were thought to control the G2/M transition. However, more recent evidence suggests that all three isoforms can dephosphorylate Cdc2/Cyclin B and play important roles in the G1/S and G2/M transitions of the cell cycle (for a complete review, see [3,4]). In Xenopus, Cdc25C most resembles human Cdc25C and is essential for promoting mitosis, while Xenopus Cdc25A does not appear to be critical for mitotic entry. This review will focus on the post-translational regulation and activation of the mitotic Cdc25C in Xenopus (hereafter referred to as Cdc25), with reference to human Cdc25C.

### Interphase Cdc25: Ser287 phosphorylation and 14-3-3 binding

During interphase, Cdc25 is inactivated by phosphorylation at Ser287 (Xenopus numbering; Ser216 in human Cdc25C) and the binding of the small acidic protein, 14-3-3. Initially, Chk1 and Chk2 (checkpoint kinases) were shown to phosphorylate Cdc25 and this phosphorylation conferred binding to 14-3-3 proteins, reportedly dampening Cdc25 catalytic activity and favoring its cytoplasmic sequestration [5-15]. This suggested that DNA-responsive checkpoints inhibited Cdc25 by phosphorylating Ser287 (discussed further below). Dunphy and colleagues were the first to report that phosphorylation and 14-3-3 binding played a role during the unperturbed cell cycle when they found that interphase Cdc25 bound to 14-3-3 and that this binding was dependent on Ser287 phosphorylation [10]. Since then, multiple kinases have been shown to phosphorylate Ser287 (Table 1). C-TAK was identified as a Cdc25-bound human kinase able to phosphorylate Cdc25C at Ser216 [16,17]. PKA was shown to negatively regulate Cdc25 on Ser287 in meiotic G2-arrested oocytes while CamKII was shown to play this role during the first mitosis in Xenopus egg extracts [18-20]. The importance of Ser287 in maintaining Cdc25's inactivation during interphase was confirmed by experiments in which Ser287 was changed to a non-phosphorylatable Ala, thereby driving premature entry into mitosis, even in the presence of DNA damage or incomplete DNA replication [10,12,14].

**Table 1:** Phosphorylated residues on Cdc25 and Wee1.

	**Xenopus Site**	**Human Site**	**+/-**	**Kinase**	**Proposed function**	**References**
**Cdc25**	T48		+	Cdc2, ERK	Enhances activity and required for Cdc2 activation, binds to Pin1	22, 29, 48, 50, 51
	T67		+	Cdc2, ?	Required for Cdc2 activation, binds to Pin1	22, 48, 50, 51
	T138	T130	+	Cdk2, ERK	Promotes 14-3-3 release, may create binding site for Plk1	21, 29, 31, 32
		S198	+	Plk1	Promotes nuclear translocation	41
	S205	S168	+/-	ERK, p38/JNK	Increases activity in Xenopus/Inhibits activity in mammalian cells	29, 54, 53
	S285	S214	+	Cdc2	Promotes PP1-mediated dephosphorylation of S287	46–48
	S287	S216	-	Chk1, Chk2, C-TAK, CamKII, PKA	Binding site for 14-3-3, blocks nuclear import	5–20, 58

**Wee1**	S38		-	Cdc2	Promotes degradation via Tome-1	83, 86
	T53		-	Cdc2	Regulates protein stability	83
		S53	-	Plk1	Promotes degradation via β-TrCP	87, 88
	Y90		+	Wee1	Promotes activity	78
	Y103		+	Wee1	Promotes activity	78
	T104		-	Cdc2	Required for Wee1 inactivation	83
	Y110		+	Wee1	Promotes activity	78
		S121	-	CK2	Promotes degradation via β-TrCP	87
		S123	-	Cdc2	Promotes Plk1 binding and degradation via β-TrCP	87, 88
	T150		-	Cdc2, ?	Required for Wee1 inactivation, binds top Pin1	83, 84
		Y295	+	Wee1	Promotes activity	77
		Y362	+	Wee1	Promotes activity	77
	S549	S642	+/-	Chk1/Akt	Promotes 14-3-3 binding and increases activity/Promotes cytoplasmic localization	58, 75–77

The effect of phosphorylation at specific residues is listed as activating (+) or inactivating (-).

### Activation of Cdc25

**Figure 1:**
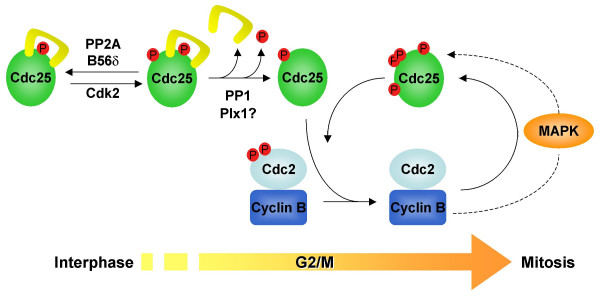
**Model of Cdc25 activation**. During interphase, Cdc25 is held inactive via by inhibitory phosphorylation at Ser287 and 14-3-3 binding. Likewise, PP2A/B56δ maintains Thr138 in the dephosphorylated state. At the G2/M transition, Cdc25 is activated in a stepwise fashion. First, Cdk2 phosphorylates Thr138 which triggers the release of 14-3-3. Phosphorylated keratin intermediate filaments assist in 14-3-3 removal from Cdc25 and Plx1 may also play a role in this process. Exposed Ser287 is then readily dephosphorylated by PP1, inducing the activation and nuclear translocation of Cdc25 and dephosphorylation of Cdc2. Once activated, Cdc2/Cyclin B phosphorylates multiple sites on Cdc25, enhancing its activity and preventing inactivation. Cdc2/Cyclin B may also activate the MAP kinase cascade which can phosphorylate Cdc25 in a parallel positive feedback loop.

Although many studies have focused on Ser287 dephosphorylation as the definitive step in Cdc25 activation, several recent lines of evidence suggest that there are multiple other critical regulatory events in the activation of Cdc25, including Thr138 phosphorylation, 14-3-3 removal from Cdc25 and the establishment of a Cdc2/Cyclin B-mediated positive feedback loop (Figure 1).

#### Thr138 phosphorylation

The activation of Cdc25 at the end of replication begins with the phosphorylation of a conserved Thr (Thr130 in human Cdc25C; Thr138 in Xenopus) in the N-terminus of Cdc25 [21]. The phosphorylation of Thr138 at the G2/M transition has been shown to be required for the release of 14-3-3 from Cdc25 [21]. Although phosphorylation of Thr138 was initially thought to be catalyzed by Cdc2/Cyclin B, this residue was later shown to be phosphorylated by another cyclin-dependent kinase, Cdk2 [21,22]. However, because Cdk2 activity is constitutive throughout the early embryonic Xenopus cell cycles and is active throughout the S and G2 phases in the somatic cell cycle, it appeared that the regulation of Thr138 phosphorylation status must lie elsewhere [23-25]. Recently, it was shown that PP2A, in association with the B56δ regulatory subunit, actively dephosphorylates Thr138 during interphase [26]. This result confirmed earlier reports that demonstrated that PP2A could inhibit Cdc25 and Cdc2/Cyclin B activation [27,28]. During replication (and under replication checkpoint conditions) Chk1 phosphorylates B56δ, promoting PP2A holoenzyme formation and the activity of PP2A/B56δ towards Thr138, holding Cdc25 inactive. Although not formally proven, it is attractive to speculate that completion of replication is accompanied by downregulation of Chk1 activity, shifting the balance between PP2A/B56δ and Cdk2, and allowing accumulation of phospho-Thr138.

Interestingly, ERK-MAP kinase has recently been shown to be able to phosphorylate Thr138 during Xenopus oocyte maturation [29]. The progesterone treatment of Xenopus oocytes, which induces oocyte maturation via activation the MAP kinase pathway, activates low levels of ERK-MAP kinase prior to Cdc2 activation and may trigger Cdc25 activation via Thr138 phosphorylation [29]. Thus, it appears that although Cdk2 may be the Thr138-directed kinase in the embryonic and somatic cell cycles, ERK-MAP kinase may play this role in progesterone-induced oocyte maturation.

#### 14-3-3 release

Once Thr138 becomes phosphorylated, 14-3-3 is released from Cdc25. Until recently, it was generally believed that 14-3-3 dissociated from Cdc25 after Ser287 was dephosphorylated due to loss of a phospho-docking site. However, this notion was contradicted by data showing that that 14-3-3 removal actually preceded Ser287 dephosphorylation and was a prerequisite for this dephosphorylation [21]. Using a mutant variant of Cdc25 that could not bind 14-3-3, but could still be phosphorylated at Ser287 (P289A) it was demonstrated that Ser287 was quickly dephosphorylated in an interphase extract, suggesting that Ser287 dephosphorylation is regulated at the level of 14-3-3 binding [30]. Although it appears that Thr138 phosphorylation can decrease the affinity of Cdc25 for 14-3-3, we do not understand the mechanistic basis for this. It is possible that phosphorylation at Thr138 creates a conformational change in the protein, which then decreases 14-3-3's affinity for Cdc25. Studies in mammalian cells suggest that phosphorylated Thr130 (human equivalent to Xenopus Thr138) provides a docking site for the binding of the polo-box domain of the polo-like kinase, Plk1 [31,32]. This suggests that Plk1 might be involved in the release of 14-3-3 from Cdc25C but this hypothesis has yet to be formally proven.

Interestingly, although Thr138 phosphorylation can decrease the affinity of 14-3-3 for Cdc25, this alone is not sufficient for 14-3-3 release from Cdc25 [26]. Using fractionated Xenopus egg extracts to look for additional factors required for 14-3-3 release, it was discovered that phosphorylated keratin 8/18 intermediate filament proteins could act as a high affinity 'sink' for binding to 14-3-3. Thus, a decrease in the affinity of 14-3-3 for Cdc25, coupled with the creation of an abundant sink for 14-3-3 binding can allow the efficient removal of 14-3-3 from Cdc25 to promote mitotic entry [26].

#### Dephosphorylation of Ser287

Once 14-3-3 is removed from Cdc25, dephosphorylation of Ser287 occurs very quickly. Initially, studies using phospho-peptide substrates incubated in Xenopus egg extracts suggested that PP2A was responsible for dephosphorylating Ser287 [33]. However, more recent work demonstrated that PP1 is the enzyme responsible for dephosphorylating Ser287 and that this dephosphorylation can only occur once 14-3-3 is released from Cdc25 [21]. Interestingly, PP1 does not employ a targeting subunit to dephosphorylate Cdc25 and a Cdc25 mutant variant that cannot bind PP1 (V105A/F107A), has wild-type 14-3-3 binding characteristics, but markedly reduced Ser287 dephosphorylation. This mutant form of Cdc25 was considerably less potent than wild-type Cdc25 in inducing entry into M-phase in Xenopus oocytes [21]. These data suggest that phosphorylation of Ser287 alone can inhibit the mitosis-promoting activity of Cdc25 independently of 14-3-3 binding and that the primary role of 14-3-3 in controlling Cdc25 activation may be to impede PP1-mediated dephosphorylation.

#### Polo-like kinase

In addition to Cdk2, the Polo-like kinase, Plx1, has been implicated in the activation of Cdc25 during the early embryonic cell cycles in Xenopus. Over a decade ago, Cdc25 was shown to be hyperphosphorylated in okadaic acid- and microcystin-treated interphase extracts that contained no detectable Cdc2 [34-36]. This suggested that there existed other kinases able to convert inactive Cdc25 into a hyperphosphorylated form that resembled mitotic, active Cdc25. In searching for these relevant kinases, Kumagai and Dunphy purified Xenopus Plx1 as a kinase that phosphorylates the N-terminus of Cdc25 [37]. Plx1 activation appeared to occur concurrently with Cdc25 and Cdc2/Cyclin B activation in Xenopus oocytes [38]. Because expression of a constitutively active Plx1 (T201D) was shown to be sufficient for activating Cdc25 and driving entry into M phase in Xenopus oocytes and extracts, Plx1 was proposed to act upstream of Cdc25 activation, while also participating in a positive feedback amplification loop to enhance Cdc25 and Cdc2/Cyclin B activation [38-40]. Consistent with a critical role for Plx1 in Cdc25 activation, both the inhibition of Plx1 kinase activity with blocking antibodies and the immunodepletion of Plx1 suppressed Cdc25 and Cdc2/Cyclin B activation in Xenopus extracts [39]. The precise mechanism underlying Plx1 activation of Cdc25 has yet to be elucidated, though, as discussed above, a role for Plx1 in promoting 14-3-3 release has been suggested.

The involvement of polo-like kinases in activating mammalian Cdc25C is more controversial. Human Plk1 has been shown to interact with mitotically phosphorylated Cdc25C and it has been proposed that Thr130 (human equivalent to Xenopus Thr138) provides a docking site for the binding of the polo-box domain of Plk1 [31,32]. Likewise, Plk1 was shown to phosphorylate human Cdc25C at a serine residue in the nuclear export signal sequence (Ser198), thus promoting nuclear import during prophase, when the nuclear envelope is still intact [41]. Despite this evidence and a demonstrated activation of Plk1 prior to Cdc2/Cyclin B activation in porcine oocytes, human cells ablated for Plk1 by RNA interference were capable of entering mitosis with high Cdc2/Cyclin B levels but failed to separate sister chromatids and complete cytokinesis [42,43]. Thus, it is unclear whether or not Plk1 is truly required for the initial activation of human Cdc25C and entry into mitosis.

### Cdc2-mediated positive feedback

Efficient entry into mitosis requires the swift and irreversible activation of Cdc2/Cyclin B. To ensure this, Cdc2/Cyclin B participates in feedback loops to control its regulators. These feedback loops create bistability, such that Cdc2 activation is like flipping a switch – it quickly switches from off to on and does not exist in an intermediate state of activation [44]. Work done in both human and Xenopus systems has demonstrated that Cdc2-catalyzed phosphorylation of Cdc25 is required for its full activation [22,36,45]. One site in particular, Ser285 (Ser214 in human Cdc25), is critical for maintaining Ser287 in the dephosphorylated state [46-48]. Mutation of this site dramatically hinders the ability of Cdc25 to promote entry into M phase in human cells and Xenopus oocytes [46-48]. Although it was originally proposed that Ser285 phosphorylation prevented rephosphorylation at Ser287 by Chk1, it has been further demonstrated that phosphorylation of this site enhances PP1's interaction with Cdc25 to maintain Ser287 in the dephosphorylated state [46-48].

Additional sites on Cdc25 that are reportedly phosphorylated by Cdc2/Cyclin B, including Thr48 and Thr67, appear to be required for the full activation of Cdc25, though this regulation appears to be independent of the status of Ser287 phosphorylation [22,48]. Kinases other than Cdc2/Cyclin B can phosphorylate Thr48 and Thr67 since okadaic acid treatment of interphase Xenopus extracts (which lack Cyclin B) can induce phosphorylation at these sites [48]. Recently, ERK-MAP kinase has been shown to phosphorylate Thr48 in Xenopus oocytes and mammalian cells [29]. Phosphorylation of Thr48 in oocytes enhances Cdc25's maturation-inducing ability and appears to, again, be a part of a positive feedback loop that involves Cdc2, Mos, MEK and ERK-MAPK [29]. Mutation of the Thr48 and Thr67 residues to alanine blocks Cdc25's ability to promote Xenopus oocyte maturation, suggesting that these sites are essential for the activation of Cdc25 [48]. It is possible that these sites directly regulate the catalytic activity of Cdc25, but it has also been reported that the WW-domains on Pin1, a peptidyl-prolyl isomerase, can bind to mitotic Cdc25 via phosphorylated Thr48 and Thr67 residues [49-51]. Pin1 binding can increase the phosphatase activity of Cdc25 phosphorylated by both Cdc2/Cyclin B and Plx1 and Pin1 binding to Cdc25 seems to be required for the maintenance of Cdc2/Cyclin B activity during mitosis [49,52].

Two other residues, Thr138 and Ser205, were originally identified as sites of Cdc2/Cyclin B-mediated phosphorylation [22]. However, as described above, Cdk2, rather than Cdc2, appears to be the kinase that initially phosphorylates Thr138 [21]. ERK-MAP kinase can then phosphorylate this site in a Cdc2-mediated feedback loop in maturing Xenopus oocytes [29]. Likewise, phosphorylation of Ser205 has been attributed to MAP kinases: ERK and p38γ in Xenopus oocytes and JNK in mammalian cells, although the effect of this phosphorylation is under debate [29,53,54]. Ser205 phosphorylation increases the activity of Cdc25 and promotes oocyte maturation in Xenopus, while the stress-induced phosphorylation of the equivalent site on mammalian Cdc25 (Ser168) has been reported to inhibit Cdc25 phosphatase activity [29,53,54]. It is possible that these reported differences are due to a difference in cellular context.

Based on these findings, it appears that Cdc2-mediated positive feedback activation of Cdc25 occurs at several levels. Following 14-3-3 release from Cdc25, PP1 binds weakly to Cdc25 resulting in inefficient dephosphorylation of Ser287. The small amount of Cdc25 that is dephosphorylated at Ser287 then dephosphorylates a small pool of Cdc2/Cyclin B, perhaps at the centrosome, before both Cdc2/Cyclin B and Cdc25 are imported into the nucleus [55]. In turn, this small pool of activated Cdc2/Cyclin B can then phosphorylate Cdc25 on Ser285, promoting high affinity binding of PP1 to Cdc25, which in turn accelerates Ser287 dephosphorylation. In tandem with these events, phosphorylation of Thr48 and Thr67 by Cdc2/Cyclin B promotes the binding of Pin1 to Cdc25, thereby enhancing its activity, further promoting the maintenance of active Cdc2/Cyclin B.

### Checkpoint regulation of Cdc25

Cdc25 is a critical target of G2/M checkpoints that ensure mitosis is not initiated until DNA is faithfully and completely replicated [56]. Checkpoint-activated kinases (Chk1 and Chk2) can catalyze the phosphorylation of Ser287, thereby enhancing 14-3-3 binding [5,6,12,14]. Mutation of Ser287 to Ala overrides both DNA-damage and replication checkpoints in fission yeast, Xenopus and human systems [10,12,15]. Interestingly, the depletion of both Chk1 and Chk2 from checkpoint activated Xenopus extracts demonstrated that 70% of the kinase activity towards Ser287 remained intact [57]. This suggested that there were other kinases that were able to phosphorylate Ser287. Indeed, as mentioned above, PKA, C-TAK, and CamKII have all been shown to phosphorylate Ser287 [17-20]. That these kinases are not regulated by DNA-responsive checkpoints suggested that there might be other loci of checkpoint control important for restraining Cdc25 activation. It also demonstrates that checkpoints maintain, rather than induce, Ser287 phosphorylation, as evidenced by a lack of obvious increase in phospho-Ser287 in response to inhibition of replication or DNA damage [58]. Given the role of 14-3-3 removal in controlling the status of Ser287 dephosphorylation, it was hypothesized that mechanisms controlling the release of 14-3-3 from Cdc25 might themselves be checkpoint regulated. Consistent with this idea, our group reported that dephosphorylation of Thr138, the site controlling the affinity of 14-3-3 binding to Cdc25, is under the control of DNA-responsive checkpoints. Specifically, when DNA replication is ongoing (or when DNA is damaged), Thr138 is maintained in a dephosphorylated state. Following DNA repair or completion of DNA replication, Thr138 dephosphorylation ensues, allowing 14-3-3 release and Cdc25 activation.

**Figure 2:**
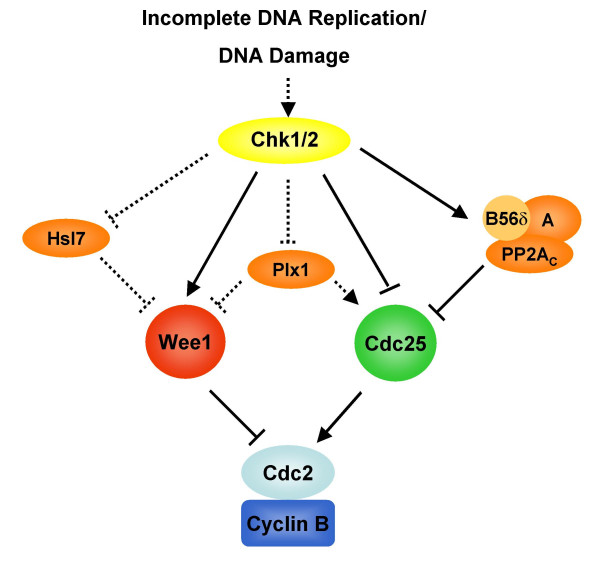
**Checkpoint regulation of Cdc2 regulators**. Prevention of Cdc2/Cyclin B activation by DNA-responsive checkpoints requires the coordinate activation of Wee1 and inactivation of Cdc25 and their regulators. Solid lines represent direct regulation while dashed lines represent proposed/indirect regulation.

Interestingly, our group found that the B56δ regulatory subunit, which directs PP2A-mediated Thr138 dephosphorylation, was a direct target of Chk1 [26]. Under checkpoint conditions, Chk1 phosphorylation of B56δ increased the activity of PP2A towards Thr138, maintaining Cdc25 in the 14-3-3 bound, inactive form. In that Chk1 depletion from egg extracts entirely abrogated B56δ phosphorylation (unlike Ser287 phosphorylation which can be catalyzed by checkpoint-independent kinases), it seems that checkpoint regulation of B56δ to control Thr138 dephosphorylation is likely a primary locus of checkpoint control [26]. Thus, checkpoint kinases control mitotic entry by suppressing Cdc25 activation via two distinct mechanisms that operate coordinately to prevent inappropriate mitosis. Checkpoints enforce the continued phosphorylation of Ser287 while activating the PP2A/B56δ phosphatase to dephosphorylate Thr138 thereby maintaining 14-3-3 binding and impeding Ser287 dephosphorylation (Figure 2).

### Wee1: background

The Wee1 family of kinases, comprised of Wee1 and Myt1, function to inhibit Cdc2/Cyclin B thus inducing interphase and preventing entry into mitosis. While both Wee1 and Myt1 can phosphorylate Tyr15 of Cdc2 in order to restrict its catalytic activity, Myt1 can also phosphorylate Thr14, which has been shown to negatively regulate Cdc2 as well [59-64]. Wee1 is a predominantly nuclear protein that prevents active Cdc2/Cyclin B from accumulating in the nucleus during interphase [65,66]. In contrast, Myt1 is membrane-bound and can both inactivate Cdc2 via phosphorylation and bind Cdc2, thus sequestering Cdc2/Cyclin B at the membrane [67,68]. Both Wee1 and Myt1 are active during interphase and become inactivated at the G2/M transition, however this review will focus on the regulation of vertebrate Wee1. In both Xenopus and mammalian systems, two isoforms of Wee1 exist, namely a maternal or embryonic isoform (Wee1A in Xenopus, Wee1B in mammals) and a somatic isoform (Wee1B/Wee2 in Xenopus and Wee1A in mammals). The maternal/embryonic isoforms are expressed in oocytes and early embryos and control the meiotic cell cycle and the early mitotic cell cycles that rapidly oscillate from interphase to mitosis [69,70]. The somatic forms of Wee1 are expressed later in development into adulthood and control the somatic cell cycle, which commences after zygotic transcription has begun [71,72]. This review will focus on embryonic Wee1 in Xenopus and somatic Wee1 in mammalian systems, which have been characterized in the greatest detail.

### Interphase Wee1: Ser549 phosphorylation and 14-3-3 binding

Like Cdc25, Wee1 is also regulated by phosphorylation and binding to 14-3-3 proteins during interphase (Table 1). Originally, a yeast two-hybrid screen identified 14-3-3ζ as a potential binding partner of the C-terminus of mouse Wee1 [73]. Subsequent analysis demonstrated that human Wee1 interacted with 14-3-3β via a C-terminal consensus motif lying between amino acids 639-646 [74]. Moreover, Wee1 protein stability and its ability to maintain interphase were enhanced by co-expression of 14-3-3β in human cells, demonstrating that 14-3-3β acts as a positive regulator of Wee1 [74]. Work in Xenopus, and then human cells, confirmed that 14-3-3 binds to Wee1 during interphase, and not mitosis, and that this binding required phosphorylation of a residue in the C-terminus, Ser549 (Ser642 in human Wee1) [75,76]. Likewise, Chk1 was shown to phosphorylate Ser549 in Xenopus Wee1 to create a phospho-binding site for 14-3-3ε and ζ, though it has been suggested that other kinases may play this role in human cells [75,76]. Regardless, a phospho-mutant of Wee1 (S549A) that was unable to bind to 14-3-3 was substantially less active than wild-type Wee1 in its ability to phosphorylate Cdc2/Cyclin B in vitro, although the mutation did not affect the intrinsic kinase activity of Wee1 [75,76]. Likewise, overexpression of the S642A mutant of Wee1 in human cells attenuated the G2 cell cycle delay observed with the overexpression of wild-type Wee1 [76]. Replacement of endogenous Xenopus Wee1 with the S549A mutant form abrogated the cell cycle delay induced by excess Chk1 [75]. Taken together, these data support the idea that Ser549 phosphorylation and 14-3-3 binding promote Wee1 activity, keeping Cdc2/Cyclin B inactive during interphase.

Recently, it was proposed that Ser642 phosphorylation and 14-3-3 binding to human Wee1 might actually inactivate Wee1 at the G2/M transition [77]. Akt was demonstrated to promote G2/M cell cycle progression by phosphorylating Ser642 of human Wee1, creating a binding site for 14-3-3θ and promoting the cytoplasmic localization of Wee1 during the late S and G2 phases of the cell cycle [77]. Likewise, the role of Ser549 phosphorylation of Xenopus Wee1 has been called into question. Unlike previous studies that utilized phospho-mutants of Wee1, Stanford and Ruderman used a phospho-specific antibody to demonstrate that Ser549 phosphorylation was low during interphase and spiked midway through mitosis in cycling Xenopus extracts when Cdc2/Cyclin B activity is highest [58]. Maintenance of Cdc2/Cyclin B activity through the addition of a non-degradable Cyclin B further enhanced Ser549 phosphorylation, suggesting the possibility of an entirely separate role for Wee1 in mitosis [58]. Together, these results suggest that we have not fully deciphered the effects of Ser549 phosphorylation and 14-3-3 binding to Wee1. It also suggests that there may be functionally different consequences for binding to different isoforms of 14-3-3, where the β, ε and ζ isoforms promote Wee1 activity, but the θ isoform serves to inactivate Wee1 via cytoplasmic sequestration. Unfortunately, we do not yet know how phosphorylation of the same residue, Ser642, by different kinases might promote the recruitment of different isoforms of 14-3-3. It is possible that the 14-3-3 isoforms are regulated directly in concert with regulation of Wee1, allowing higher affinity interactions of particular 14-3-3 isoforms under particular physiological conditions.

Wee1 is also positively regulated by autophosphorylation during interphase (Table 1). Three sites on Xenopus Wee1 (Tyr90, Tyr103 and Tyr110) and two sites on human Wee1 (Tyr295 and Tyr362) have been identified as sites of autophosphorylation that promote Wee1 activity [77,78]. Interestingly, the mutation of these sites on Xenopus Wee1 to nonphosphorylatable residues did not affect the ability of Wee1 to phosphorylate Cdc2 in vitro. However, mutation compromised the kinase in its ability to inhibit M-phase in progesterone treated oocytes, further suggesting that autophosphorylation during interphase promotes Wee1 kinase activity [78].

### Inactivating Wee1 at the G2/M transition

Research over the last decade has demonstrated that the activation of Cdc25 consists of many regulated steps that proceed in a tightly coordinated manner. Unfortunately, the inactivation of Wee1 is less well understood. Both the human and Xenopus Wee1 kinases are negatively regulated by phosphorylation at the G2/M transition (Table 1) [79-82]. Initially, yeast and Xenopus Wee1 were shown to become highly phosphorylated at the N-terminus in M-phase, which correlated with a loss in activity [80,81]. Further investigation showed that part of the electrophoretic mobility shift in Wee1 at M-phase was due to Cdc2-mediated phosphorylation [80]. However, depletion of Cdc2 from M-phase Xenopus egg extracts demonstrated that other kinases with the ability to phosphorylate Wee1 during M-phase remained following this depletion [80]. Interestingly, treatment of interphase Xenopus extracts with the phosphatase inhibitor, okadaic acid, creates a similar electrophoretic shift in Wee1 mobility as seen in mitosis [81]. This again strongly suggests that kinases other than Cdc2/Cyclin B can phosphorylate Wee1, however it is not known if any of these sites get phosphorylated prior to the activation of Cdc2/Cyclin B. Kim *et al*. recently used MALDI-TOF mass spectrometry to identify multiple mitotic phosphorylations on Xenopus Wee1 [83]. Two sites, Thr104 and Thr150, were identified as being critical for the downregulation of Wee1 activity in M-phase. Interestingly, Wee1 bearing mutations at these sites inhibited the mitotic activation of Cdc2/Cyclin B more effectively than wild-type Wee1. Moreover, Thr150 became phosphorylated prior to nuclear envelope breakdown [83]. Recently, the Thr150 equivalent in Xenopus Wee1B, Thr186, has been shown to be required for the Cdc2-mediated downregulation of Wee1B kinase activity at M phase (discussed further below) [84]. Thus, Thr150 appears to be involved in the Cdc2-mediated negative regulation of Wee1 but these results potentially place the phosphorylation of Thr150, which is also conserved in other species of embryonic and somatic Wee1, upstream of Cdc2/CyclinB activation, although it does not rule out its role in a Cdc2-mediated negative loop. The precise functional consequences of Thr104 phosphorylation remain to be elucidated.

Aside from being downregulated by phosphorylation at the G2/M transition, it has long been proposed that Wee1 is also regulated by proteolytic degradation [82,85]. There was 10-fold less Wee1 in mitotically-arrested human cells as compared to Wee1 protein levels in G2 cells [82]. This decrease in total Wee1 protein was attributed to degradation as inhibition of protein synthesis did not affect the decrease in protein seen at mitosis [82]. Likewise, it was reported that embryonic Xenopus Wee1 was also degraded intranuclearly at the G2/M transition [85]. Degradation of embryonic Xenopus Wee1 can occur even if Cdc2 activity is inhibited, suggesting that it may be a primary mode of Wee1 regulation upstream of Cdc2/Cyclin B [85]. However, available evidence suggests that degradation of human somatic Wee1 represents a Cdc2-mediated negative feedback mechanism; this will be discussed further below [80,81].

Although we know that hyperphosphorylation of Wee1 correlates with its inactivation, we still do not fully understand the molecular requirements for inactivation of Wee1. Thr138 phosphorylation of Cdc25 and the subsequent release of 14-3-3 are currently the most upstream events known to be required for the activation of Cdc25. Interestingly, it has yet to be shown whether Wee1 inactivation requires similar steps. For example, it may be that additional sites (known or unknown) on Wee1 must be phosphorylated to release 14-3-3 and inactivate Wee1. Could it be Thr150? Do Cdk2 and PP2A/B56δ regulate this potential site? That Wee1 becomes hyperphosphorylated and presumably inactivated by okadaic acid, suggests that either Wee1 or the kinases that inactivate Wee1 are directly regulated by the activity of a phosphatase during interphase. In addition, although 14-3-3 does not bind to Wee1 during mitosis (despite phosphorylation of its docking site, Ser549), is the release of 14-3-3 from Wee1 a requirement for entry into mitosis? Is Ser549 dephosphorylation a requirement for entry into mitosis and what phosphatase is responsible for the dephosphorylation? Are Ser549 phosphorylation and 14-3-3 binding dependent on one another or regulated separately? Taking a cue from Cdc25 studies, it will be interesting to determine whether the intermediate filament phospho-sink required for 14-3-3 removal from Cdc25 is also required for the release of 14-3-3 from Wee1.

### Cdc2-mediated negative feedback and degradation

Just as Cdc2/Cyclin B phosphorylates Cdc25 to enhance its activation, Cdc2-mediated phosphorylation of Wee1 during M-phase was initially reported many years ago, however its consequences remained elusive [80]. After it was reported that nuclear Wee1 protein was degraded after the completion of S phase in Xenopus and that this degradation was dependent on the Cdc34 E2 enzyme, Ayad *et al*. identified Tome-1 (Trigger of Mitotic Entry) as a novel SCF-type E3 ubiquitin ligase required for the degradation of Wee1 and mitotic entry [85,86]. Tome-1 recognition of Wee1 is dependent on the phosphorylation of Ser38, which was identified as a Cdc2-directed SP site found only on embryonic Xenopus Wee1 [86]. Mutational analysis demonstrated that Thr53 in Xenopus Wee1 also regulates the stability of Wee1 and both Ser38 and Thr53 contribute to Wee1's electrophoretic mobility shift at mitosis, but again, these sites are poorly conserved across species [83]. Recently, it was reported that phosphorylation of human Wee1 at Ser123 by Cdc2/Cyclin B initiates a cascade leading to Wee1 degradation as well [87,88]. These findings demonstrate that Cdc2 can initiate a negative feedback loop to downregulate Wee1. Phospho-Ser123 was shown to directly interact with the WD40 repeat domain of the E3 ubiquitin ligase, β-TrCP [87]. Phosphorylation of Ser123 by Cdc2/Cyclin B also promotes the phosphorylation of Ser121 by CK2, which creates another β-TrCP binding site [87]. Furthermore, phospho-Ser123 was shown to provide a docking site for the Polo-like kinase, Plk1, whose phosphorylation of Wee1 at Ser53 creates a third phosphodegron, also recognized by β-TrCP [87,88]. Although the Plk1 homolog in budding yeast, Cdc5, had been shown to interact with and negatively regulate yeast Wee1, this was the first example of negative regulation of Wee1 by Plk1 in higher eukaryotes [89]. Thus, Cdc2/Cyclin B, CK2 and Plk1 appear to act in concert to promote the degradation of human Wee1 at the G2/M transition.

Recent work on the somatic isoform of Xenopus Wee1, Wee1B, has described another mechanism for the Cdc2-mediated inactivation of Wee1 kinase activity at M phase [84]. First, the authors identified a conserved region in the N-terminal regulatory domain of Wee1B, termed the Wee-Box, which positively regulates the catalytic domain of Wee1B during interphase. In mitosis, a conserved Thr within this domain (Thr186 in Xenopus Wee1B, Thr150 in Xenopus Wee1A) is phosphorylated by Cdc2/Cyclin B, creating a binding site for Pin1, which is required for the inactivation of the kinase at M phase [84]. Previously, Pin1 was shown to bind to mitotically phosphorylated Wee1 yet until now, the phospho-binding site and functional consequences of Pin1 binding had not been elucidated [51].

### Checkpoint regulation of Wee1

As checkpoint pathways function to inhibit the activation of Cdc25, they also act to ensure the continued suppressive effects of Wee1 (Figure 2). Genetic analyses in yeast were the first to suggest that Wee1 was a target of G2/M checkpoints [56,90,91]. Subsequently, Michael and Newport were able to show that nuclear Wee1 protein stability was controlled by DNA-responsive checkpoints [85]. Following the inhibition of replication or DNA damage in Xenopus extracts, Wee1 protein is stabilized, whereas during the time of mitotic entry Wee1 was found to be degraded intranuclearly [85]. In addition, it was found that a Wee1-interacting protein, Hsl7, originally shown to regulate the stability of the S. cerevisiae Wee1 homolog (Swe1) in response to the morphogenesis checkpoint, could promote the intra-nuclear degradation of embryonic Xenopus Wee1 at the G2/M transition [92,93]. Moreover, overexpression of Hsl7 to promote Wee1 degradation inappropriately could override a DNA replication checkpoint to trigger mitotic entry, while depletion of Hsl7 promoted Wee1 stabilization and cell cycle arrest prior to mitotic entry [93]. It is noteworthy that Hsl7-mediated Wee1 degradation did not depend upon Cdc2 activity, consistent with a possible role for Hsl7 in checkpoint pathways acting upstream of Cdc2/Cyclin B. Together, these results suggest that promoting Wee1 stability is integral to maintaining an interphase state when DNA-responsive checkpoints are activated.

Phosphorylation of Ser549 also appears to be regulated by checkpoints. Early results in cycling Xenopus extracts, showed that replacing endogenous Wee1 with the S549A mutant attenuated the cell cycle delay induced by excess Chk1 or the DNA replication checkpoint [75]. Likewise, using a phospho-specific antibody directed against phosphorylated Ser549, it was shown that checkpoint activation induced an increase in phosphorylated Ser549, accompanied by a slight increase in Wee1 kinase activity towards Cdc2 [58]. Although Stanford and Ruderman reported little phosphorylation of Ser549 in an unperturbed interphase, they clearly saw an increase in phosphorylation when a replication checkpoint or DNA damage checkpoint were induced or when PKA, which blocks mitotic entry, was added to extracts [58]. Dynamic regulation of Ser549 phosphorylation under checkpoint conditions again suggests that Wee1 might be regulated differently from Cdc25, where Ser287 phosphorylation is maintained but not upregulated by checkpoints. The identification of a highly conserved site in the N-terminus of Wee1, Thr150, that becomes phosphorylated in M-phase (and potentially prior to Cdc2/Cyclin B activity) suggests that there may also be other sites controlled by DNA-responsive checkpoints [83].

## Conclusion

Preventing premature entry into mitosis while DNA replication is ongoing or under conditions of DNA damage, not only involves suppressing mitotic activators, such as Cdc25, but also involves the active upkeep of interphase-promoting factors, such as Wee1. Cells achieve this feat via mechanisms that help inhibit the activators and activate the inhibitors of mitosis. Chk1 phosphorylation of Wee1 and Cdc25 creates a 14-3-3 binding site on each protein during interphase, affecting either the enzymatic activity or localization of Wee1 and Cdc25. Multiple kinases that have been shown to activate Cdc25 have also been shown to inactivate/degrade Wee1 at the G2/M transition. Aside from Plx1, PKA has been shown to maintain the G2 meiotic arrest in mammalian oocytes by the concurrent activation of the embryonic isoform of mammalian Wee1, Wee1B, and inactivation of Cdc25 [18,20,70]. Once Cdc2/Cyclin B is activated, Cdc2-mediated feedback loops, both positive and negative, ensure that the activators stay active and the inhibitors are inhibited – exemplifying the perfectly engineered bistable switch [44,94]. Cdc2-mediated phosphorylation of Cdc25 ensures dephosphorylation of Ser287 and nuclear import of Cdc25, while Cdc2-mediated phosphorylation of Wee1 assists in the destruction of the protein. Through the concerted action of regulators that both inhibit Wee1 and activate Cdc25, cells can achieve a sharp, swift transition into mitosis. Conversely, through inhibition of these regulatory pathways, DNA-responsive checkpoints can ensure that mitosis does not occur prematurely in the presence of incompletely replicated or damaged DNA.

## Competing interests

The author(s) declare that they have no competing interests.

## Authors' contributions

JAP and SK both drafted the manuscript.
